# Le tetanos chez le grand enfant dans un hôpital pédiatrique à Yaoundé, Cameroun

**Published:** 2012-02-29

**Authors:** Mina Ntoto Njiki Kinkela, Félicitée Nguefack, Hubert Mbassi Awa, David Chelo, Dominique Enyama, Marie Mbollo Kobela, Paul Olivier Koki Ndombo

**Affiliations:** 1 Centre Mère et Enfant (CME) de la Fondation Chantal BIYA de Yaoundé, Cameroun; 2 Faculté de Médecine et des Sciences Biomédicales de Yaoundé, Cameroun; 3 Ministère de la santé Publique ; Secrétariat Permanent du Programme Elargi de Vaccination, Yaoundé, Cameroun

**Keywords:** Tétanos, vaccination, rappels vaccinaux, grands enfants, Cameroun

## Abstract

Le tétanos est évitable par la vaccination, mais peut survenir en cas d'une immunisation incomplète. Nous avons mené une étude sur les dossiers médicaux des enfants admis pour tétanos entre 2008-2009 au Centre Mère et Enfant de la Fondation Chantal BIYA à Yaoundé. Le but était d'analyser les circonstances de survenue et les manifestations cliniques du tétanos chez le grand enfant, afin de proposer des stratégies de prévention adaptées au contexte camerounais. Le statut vaccinal était inconnu chez un patient, les autres (80%) n'avaient pas reçu de rappel vaccinal. Les portes d'entrée étaient les plaies aux membres, l'une était secondaire à une injection médicamenteuse. Tous ont présenté le tétanos généralisé. Le décès était survenu chez un patient. Le tétanos n'est pas rare chez le grand enfant au Cameroun. Il se dégage ainsi la problématique des rappels vaccinaux.

## Introduction

Le tétanos est une affection provoquée par la tétanospasmine, produite par le *Clostridium tetani*. Cette toxine sécrétée à partir d′une plaie contaminée par ce bacille ubiquitaire, provoque une fois migrée au niveau des cornes antérieures de la moelle épinière et du cervelet, une augmentation du tonus musculaire. Sa durée d'incubation dépend de la quantité de toxines formées. Elle est brève dans les cas graves, et plus longue dans les cas bénins [1]. Nous avons mené une étude dont but était d'analyser les circonstances de survenue, les manifestations cliniques, les modalités thérapeutiques et l’évolution clinique du tétanos chez l'enfant au-delà de la période néonatale. Le diagnostic du tétanos reposait sur la présence du trismus associé à des contractures paroxystiques. Les autres éléments anamnestiques étaient la recherche d'une effraction cutanée ainsi que les antécédents vaccinaux antitétaniques des patients. Un enfant était dit correctement vacciné s'il avait reçu ses trois doses de vaccin antitétanique combiné aux vaccins contre la diphtérie et la coqueluche aux âges recommandés par le programme national de vaccination. Seuls les cas survenus au-delà de la période néonatale étaient retenus dans cette étude. Le score de Dakar avait servi à la classification des cas afin de déterminer la sévérité de la maladie.

## Observations

### Cas N° 1

Fille de 8 ans domiciliée à Akonolinga (région du centre-Cameroun), référée dans notre formation sanitaire pour la prise en charge d'un « syndrome tétanique ». En effet, une semaine avant, ses parents avaient constaté chez elle des troubles de la marche notamment, des chutes et des contractures musculaires des membres. Ils avaient alors consulté à l'hôpital de district de leur localité. Au décours de la consultation dans ledit hôpital, il avait été noté une lésion ulcérée siégeant au niveau du poignet droit faisant évoquer un ulcère de Buruli. Sur cette lésion qui était survenue un mois auparavant, les parents appliquaient un mélange de décoctions traditionnelles. Les antécédents de la patiente révélaient qu'elle avait bénéficié de tous les vaccins du programme élargi de vaccination dans le bas âge, mais le carnet de vaccination n’était pas disponible. A l'admission dans notre centre, son état général était altéré, mais la conscience était normale. Ses paramètres vitaux étaient normaux. Son état nutritionnel était satisfaisant avec un poids de 27 kg (correspondant au 75e percentile). Sa température était de 38,5°C. L'examen physique notait par ailleurs un trismus, des spasmes provoqués sur un fond de contractures généralisées. Au niveau du poignet droit on notait une plaie sur laquelle étaient appliqués des cataplasmes traditionnels. Elle avait été admise dans l'unité de soins intensifs où la prise en charge avait consisté en la pose d'une sonde nasogastrique pour l'alimentation. Elle avait été isolée de la lumière et des bruits. Elle avait également bénéficié d'un traitement associant une sérothérapie antitétanique (3000 UI sous cutanée, de la pénicilline G à 100 000 UI/Kg/jour et du diazépam à 1 mg/Kg toutes les 6 heures par voie intraveineuse. Un parage et un lavage de la plaie à l'eau oxygénée avaient été faits. L’évolution du tétanos avait été favorable. Au bout de 7 jours, les crises de spasme disparaissaient et les contractures diminuaient, mais l'ulcère n'a pas cicatrisé. Sa sortie de l'hôpital était autorisée au 17e jour après l'administration d'un vaccin antitétanique. Elle a gardé les séquelles résiduelles à type d'enraidissement de la colonne vertébrale pour lesquelles une rééducation a été envisagée en vue de rétablir la mobilité normale du rachis. La prise en charge de sa plaie devait être poursuivie dans sa localité dans un centre spécialisé pour le traitement de l'ulcère de Buruli. Les parents joints par téléphone après six semaines, on affirmé que le rappel du vaccin avait été fait et que la cure chirurgicale de la lésion était réalisée. En conclusion, il s'agissait d'un tétanos survenu sur un ulcère négligé, coté au stade I de la classification de Dakar chez une enfant n'ayant pas reçu de rappel vaccinal.

### Cas N° 2

Garçon de 7 ans domicilié à Yaoundé, admis dans notre centre, 2 jours après le début d'une symptomatologie marquée par des douleurs thoraciques à type de constriction suivies de contractures généralisées. Il avait été référé d'un centre de santé qui avait initié un traitement comprenant de l'ampicilline, noramidopyrine, tiémonium, dexamethasone, diclofénac, acide niflumique et de la quinine en perfusion. Les contractures s’étaient aggravées sous ce traitement. Ses antécédents ne révélaient rien de contributif. Sur le plan immunologique, il aurait été vacciné normalement jusqu’à l’âge de 9 mois et n'avait pas reçu de rappel à 18 mois. A l'admission sa conscience était conservée, sa température de 37,5°C, le rythme cardiaque et la fréquence respiratoire étaient dans les limites de la normale. Sur le plan nutritionnel, son poids était de 20 Kg (10e percentile). A l'examen physique, l'on notait un trismus, des spasmes en réponse aux stimuli douloureux, des contractures généralisées et une cicatrice de plaie survenue une semaine auparavant sur la plante du pied gauche. Il avait été admis dans l'unité de soins intensifs et avait bénéficié d'un traitement fait de sérum antitétanique de pénicilline G, diazépam, et de l'alimentation par gavage. L’évolution était rapidement favorable, marquée par la diminution des contractures. L'ouverture de la bouche était possible le troisième jour et l'alimentation orale effective. La sortie était faite au bout de 10 jours et le vaccin antitétanique devait se faire en ambulatoire. Les suites post hospitalisation étaient normales, il a en outre bénéficié de la vaccination antitétanique et du rappel. Nous avions conclu à une forme frustre de tétanos généralisé de l'enfant au stade I de la classification internationale de Dakar.

### Cas N° 3

Garçon de 13 ans résidant à Yaoundé, hospitalisé, deux jours après le début des symptômes marqués par des douleurs cervicales et lombaires, et une contracture des masséters. L'anamnèse retenait la notion d'une plaie traumatique sur la jambe gauche survenue 10 jours auparavant. En automédication, de la povidone iodée et de la poudre de benzathine pénicilline étaient appliquées sur cette plaie. Par ailleurs, le patient recevait de l’érythromycine à raison de 1g par jour depuis 5 jours. A l'admission dans notre formation sanitaire, il était conscient, sa température était de 37°8 C. Sur le plan nutritionnel, son poids était de 37 Kg (situé entre le 10e et le 25e percentile). L'examen avait objectivé le trismus, des spasmes assez fréquents sur un fond de contracture généralisée. Il avait été admis dans l'unité de soins intensifs et sa prise en charge avait consisté en l'administration du sérum antitétanique, du diazépam, de la pénicillinothérapie, au parage de la plaie et à l'isolement de la lumière et des bruits. Les besoins nutritionnels avaient été assurés par des liquides par voie intraveineuse et par une alimentation précoce par sonde nasogastrique. L’évolution était marquée par la persistance et même l'aggravation des spasmes très douloureux, qui n'avaient cédé que sous des doses très élevées de diazépam (8 mg/Kg de poids corporel/jour) et l'adjonction du paracétamol injectable contre les douleurs. Il avait bénéficié de la vaccination antitétanique avant sa sortie autorisée au 19e jour d'hospitalisation. Ce malade avait gardé des séquelles et devait bénéficier d'une kinésithérapie motrice pour la raideur de la colonne vertébrale. Le rappel vaccinal a été fait conformément à la prescription, selon les informations recueillies des parents joints par téléphone. Nous avons conclu à une forme de tétanos du stade I de la classification de Dakar dont la porte d'entrée était la plaie mal soignée.

### Cas N° 4

Il s'agissait d'une fillette de 4 ans résidant à Yaoundé avec une notion d'injection intramusculaire de quinine. Son statut vaccinal était ignoré des parents. Elle était admise un jour après le début de la symptomatologie marquée par des contractures et des difficultés à ouvrir la bouche survenues 8 jours après une injection de quinine. L'examen physique initial notait une température de 39.5°C, une altération de la conscience (score de Blantyre à 3/5). Son état nutritionnel était satisfaisant, notamment son poids était de 16,5 Kg (situé entre 50e et le 75e percentile). Un trismus, des spasmes très fréquents et une contracture des membres et du tronc étaient également observés. Par ailleurs, on notait une inflammation de la cuisse gauche dont la circonférence était de 39,3 contre 37 cm du côté droit. Elle avait été admise dans l'unité de soins intensifs où elle avait été isolée des bruits et de la lumière. Sa prise en charge comportait par ailleurs du sérum antitétanique, du diazépam, de la pénicillinothérapie, des pansements alcoolisés de la cuisse et des perfusions de solutés et une alimentation par sonde nasogastrique. L’évolution avait été rapidement défavorable marquée par une aggravation en quelques heures de la fréquence et de l'intensité des spasmes, de l'attitude en opisthotonos et des troubles végétatifs à type d'hypersudation, et de la persistance d'une fièvre à 39.5°C. Le bilan infectieux envisagé notamment, les hémocultures et l'hémogramme n'ont pu être faits. Le décès survenait12 heures après l'admission. Nous avons conclu à un tétanos iatrogène au stade II de la classification internationale de Dakar.

En résumé, les quatre malades avaient présenté la forme généralisée du tétanos. Trois d'entre eux auraient bénéficié de la série des vaccins du programme élargi de vaccination et sans rappel vaccinal. Le statut vaccinal était inconnu chez le quatrième. Dans 75% des cas la porte d'entrée était la plaie négligée sur un membre, dont un ulcère de Buruli. Un cas était iatrogène à la suite d'une injection probablement septique, et d’évolution rapidement mortelle. L'invasion était très rapide dans un cas chez qui le décès est survenu quelques heures après l'admission. La durée moyenne d'hospitalisation était de 11,8 jours avec des extrêmes allant de 12 heures à 19 jours.

## Discussion

Dans les pays développés, l'incidence du tétanos est devenue très faible du fait de la politique vaccinale qui intègre les rappels, de l'amélioration des conditions d'hygiène et la prise en charge correcte des plaies [2]. Par contre dans les pays en voie de développement, le tétanos demeure un problème de santé majeur. Au Cameroun, malgré le renforcement de la mise en oeuvre des stratégies en vue de l’élimination du tétanos maternel et néonatal, cette affection continue de faire beaucoup de victimes. Avec une couverture vaccinale en diphtérie-tétanos-coqueluche (troisième dose) de 82% en 2007, 146 cas de tétanos avaient été notifiés au Cameroun dont 47 cas chez le nouveau-né [3].

Le tétanos est une maladie à déclaration obligatoire. En dehors des études menées dans certains centres hospitaliers, son épidémiologie est insuffisamment maitrisée chez le grand enfant dans les pays en voie de développement où la surveillance est limitée à celle du tétanos maternel et néonatal. A Dakar la prévalence hospitalière était de 5,3% chez les enfants âgés de 1 à 15 ans, dont un taux de létalité de 8% [4].

La moyenne d’âge dans notre série était de 8 ans, les extrêmes allaient de 4 à 13 ans, proche de la moyenne de 8,8 ans retrouvée dans l’étude réalisée à Dakar [4]. Certains de ces patients auraient échappé aux stratégies vaccinales utilisées pour les atteindre. Même après avoir bénéficié de la série de vaccination primaire, l'immunisation n'est pas garantie dans certaines conditions. Galazka a cité en 1993 certains auteurs ayant rapporté dans leurs études, des cas de tétanos survenus chez des malades vaccinés dans l'enfance et n'ayant pas bénéficié de rappels vaccinaux [5]. Trois des 4 patients faisant partie de notre étude avaient reçu les trois doses de vaccins primaires, mais n'avaient pas eu de rappels. L'immaturité immunologique limiterait la persistance des anticorps acquis par la vaccination primaire dans l'enfance [6]. Par ailleurs, l'immunité contre le tétanos décline avec le temps chez l'enfant vacciné, si bien qu'entre 10 et 16 ans, environ un cas sur cinq ne possède plus d'anticorps protecteurs [7]. Pour cette raison, les rappels vaccinaux sont recommandés afin d’établir la protection durable contre le tétanos. L'OMS recommande que les pays ayant atteint une couverture élevée pour la série de vaccination primaire fassent une ou deux doses de rappel de vaccin antitétanique durant l'enfance [8]. Cette recommandation serait illusoire au Cameroun où la couverture vaccinale antitétanique reste en deçà des objectifs du programme élargi de vaccination (PEV), soit 88% pour la troisième dose du vaccin pentavalent. Elle était de 81,5% en janvier 2011[9]. La problématique de la politique des rappels vaccinaux demeure au Cameroun. En effet, le programme national de vaccination assure la couverture gratuite en vaccins chez les enfants de 0 à 11 mois et des femmes âgées de 15 à 49 ans contre le tétanos. Chez les enfants, cette période est interprétée à tort par des professionnels de santé et même les populations comme le niveau d'achèvement des vaccins.

L'immunité naturelle n'existe pas dans le tétanos. La porte d'entrée est la plaie mal vascularisée, souillée de terre ou de débris dans lesquels le bacille de Nicolaïer peut trouver un milieu favorable pour se développer et produire sa toxine neurotrope. Les injections intramusculaires peuvent aussi faire le lit du tétanos, des cas similaires ont été rapportés à Abidjan en Côte d'ivoire et à Dakar [10].

Pour cette raison, certains auteurs pensent que la prévention du tétanos doit faire partie de la prise en charge des plaies profondes. En cas de plaie à risque, la vaccination antitétanique est indiquée chez tout enfant de moins de 6 ans non vaccinés correctement, ou toute personne âgée de 7 ans et plus ayant reçu trois doses de vaccins primaires, dont la dernière dose remonte à plus de 10 ans. Elle est également recommandée en cas d'incertitude sur le statut vaccinal. Cette vaccination supplémentaire ne serait pas réalisable chez tous les enfants camerounais dont certains sont issus des familles qui vivent avec moins d'un dollar par jour. Au-delà de 11 mois, les charges financières liées aux vaccins reviennent aux parents, sauf en contextes particuliers (activités de vaccination supplémentaires, Semaine d'Actions de Santé et de Nutrition Infantile et Maternelle, stratégie de Prise en charge Intégrée des Maladies de l'enfant(PCIME). Il en est ainsi des rappels vaccinaux.

Grâce aux mesures préventives, la mortalité liée au tétanos a diminué dans les pays développés, mais reste élevée dans les pays en voie de développement. En milieu hospitalier africain il était de 38,9% à Kigali, [11]. L'insuffisance respiratoire serait la principale cause de décès chez ces patients. Dans certaines études, les patients ont bénéficié d'une trachéotomie et d'une ventilation mécanique [11]. Cette réanimation aurait trouvé une indication chez ce patient dont le décès avait été enregistré dans notre série. Par ailleurs il n'est pas exclu qu'un sepsis ait été associé au tableau clinique de cet enfant. Nous n'avons pas pu l'exclure du fait de l'insuffisance de moyens financiers des parents, du manque de la permanence des bouillons pour les hémocultures et de la rapidité de survenue du décès. Sa prise en charge s’était limitée au traitement symptomatique des spasmes, à l'alimentation, aux perfusions de solutés, au parage de la plaie et à l'antibiothérapie. Les gestes invasifs sont rarement appliqués dans notre contexte. La pénicillinothérapie, mais surtout le parage de la plaie souillée sont essentiels pour permettre l’éviction du germe. Ce traitement devrait être soutenu par l'administration du vaccin antitétanique avant la sortie de l'hôpital au risque de conduire à une occasion manquée pour la vaccination. Ce qui n'est pas toujours facile à réaliser chez les enfants hors cible du PEV dans notre contexte.

## Conclusion

Au Cameroun, le tétanos n'est pas rare chez les enfants âgés de plus d'un an. Trois malades inclus dans cette étude étaient initialement vaccinés, mais n'avaient reçu aucun rappel après la 3ème dose de DTCoq. Ceci pose la problématique de la politique nationale des rappels de la vaccination anti tétanique chez les enfants hors cible du PEV. Une amélioration de la couverture vaccinale en général et des rappels en particulier s'impose à travers le renforcement des capacités du personnel de santé au Cameroun. Les parents doivent être sensibilisés sur la nécessité de poursuivre la vaccination des enfants au-delà de 11 mois en attendant l'adoption des rappels vaccinaux dans le PEV. Tout enfant non vacciné correctement et ayant une plaie susceptible d’être tétanigène doit faire l'objet des soins préventifs contre le tétanos. La plaie doit être nettoyée soigneusement, et l'administration prophylactique du sérum ainsi que le vaccin antitétanique doit être indiquée chez toute personne susceptible de faire le tétanos.
